# Charge-Separated Reactive Intermediates from the UV Photodissociation of Chlorobenzene in Solution

**DOI:** 10.1021/acs.jpca.2c05327

**Published:** 2022-09-23

**Authors:** Min-Hsien Kao, Andrew J. Orr-Ewing

**Affiliations:** School of Chemistry, University of Bristol, Cantock’s Close, Bristol BS8 1TS, United Kingdom

## Abstract

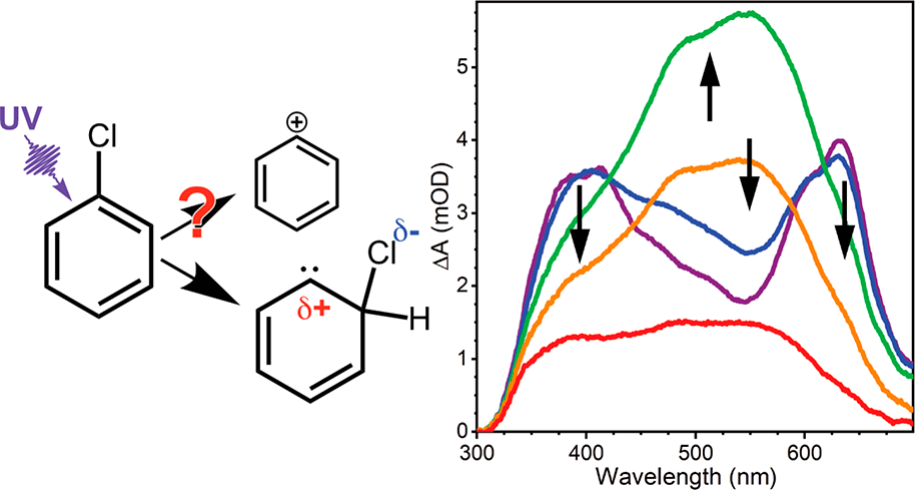

Although ultraviolet (UV)-induced photochemical cleavage of carbon–halogen bonds in gaseous halocarbons is mostly homolytic, the photolysis of chlorobenzene in solution has been proposed to produce a phenyl cation, c-C_6_H_5_^+^, which is a highly reactive intermediate of potential use in chemical synthesis and N_2_ activation. Any evidence for such a route to phenyl cations is indirect, with uncertainty remaining about the possible mechanism. Here, ultrafast transient absorption spectroscopy of UV-excited (λ = 240 and 270 nm) chlorobenzene solutions in fluorinated (perfluorohexane) and protic (ethanol and 2,2,2-trifluoroethanol) solvents reveals a broad electronic absorption band centered at 540 nm that is assigned to an isomer of chlorobenzene with both charge-separated and triplet-spin carbene character. This spectroscopic feature is weaker, or absent, when experiments are conducted in cyclohexane. The intermediate isomer of chlorobenzene has a solvent-dependent lifetime of 30–110 ps, determined by reaction with the solvent or quenching to a lower-lying singlet state. Evidence is presented for dissociation to *ortho*-benzyne, but the intermediate could also be a precursor to phenyl cation formation.

## 1 Introduction

Aryl cations are some of the most reactive intermediates used in organic synthesis.^1^ They can be produced by thermal decomposition of an aryl diazonium compound, and their reactions include intramolecular cyclization or hydride transfer followed by hydrolysis.^2−7^ The cyclization reactions can bind adjacent oxygen or sulfur atoms to form alkyldibenzofuranium and alkyldibenzothiophenium species.^8^ The phenyl cation, c-C_6_H_5_^+^, contains a charged benzene ring and has potential use in molecular nitrogen capture to afford diazonium compounds, as observed by mass spectrometry.^9,10^ If scalable, this N_2_-capture reaction could provide an alternative to the energy-demanding Haber–Bosch process for extraction of nitrogen from the atmosphere to prepare chemical feedstocks.^11^ The product diazonium compound can be used for the synthesis of azobenzene, which has been targeted for use in liquid crystals, photochemical molecular switches, and antibiotics.^12−14^

The phenyl cation has singlet and triplet spin-state forms. The singlet phenyl cation is closed shell (π^6^σ^0^) and is more stable than the open-shell triplet phenyl cation (π^5^σ^1^) by ∼100 kJ mol^–1^ when there are no substituent groups attached.^15,16^ The most obvious difference in the geometries of the two forms is the bond angle about the carbocation center (i.e., the C atom formally carrying the positive charge). In the singlet phenyl cation, this ring bond angle is about 147°, whereas in the triplet phenyl cation, it is about 125° which is closer to the angle found in a phenyl radical, leading to the suggestion that the triplet state is an intermediate to the formation of the singlet phenyl cation.^15,17^ In contrast, the triplet aryl cation is usually more stable than the singlet when an electron-donating group is attached on *para-* or *ortho-*sites of the phenyl ring.^18,19^ Because of their different electronic configurations, the chemo-selectivities of the two forms of phenyl cations are not the same. The singlet phenyl cation is a strong electrophile, whereas the triplet phenyl cation selectively reacts with π-nucleophiles like alkynes and aromatic compounds.^1,20−22^ The mechanism for nitrogen capture by the singlet phenyl cation is similar to nitrogen fixation by boron, which is based on σ donation from N_2_ to an empty σ orbital and π back-donation to the empty π* orbital of N_2_.^23,24^

Recent reports suggest that phenyl cations can be conveniently generated from UV photodissociation of phenyl halides such as chlorobenzene,^1,25^ in competition with the well-known homolytic C–X (X = halogen) bond fission of organohalides to make radical fragments.^26−30^ The current work explores this proposition for solutions of chlorobenzene in various solvents. Previous experimental studies of the photochemistry of gas-phase chlorobenzene are supported by quantum chemical calculations of excited-state C–Cl bond dissociation pathways.^28,29,31^ For example, Liu et al. computed ground- and excited-state potential energy surfaces for chlorobenzene along the C–Cl bond extension coordinate, using multireference complete active space self-consistent-field second-order perturbation theory (MSCASPT2).^29^ These calculations show that ultraviolet (UV) excitation at 193 nm causes direct, homolytic bond cleavage because the S_4_ (nσ*) state reached is dissociative and barrierless.^29,32,33^ Photodissociation has also been reported at wavelengths from 248–266 nm, corresponding to initial population of the S_1_ (ππ*) state.^27,28,30,31^ Because the S_1_ state is bound, the dissociation occurs after intersystem crossing (ISC) or internal conversion (IC) to other electronic states. Crossings to the dissociative S_4_ (nσ*) and T_5_ (nσ*) states are accessible from the S_1_ state for excitation wavelengths of 248 nm or less. However, at 266 nm the photon energy is insufficient to reach the conical intersections or crossings to these repulsive states, so slower photodissociation instead follows the IC, which populates vibrationally excited levels of the S_0_ electronic state lying above its dissociation asymptote. Time-resolved mass spectrometry revealed two time constants for relaxation of 266 nm photoexcited chlorobenzene, which were assigned to intramolecular vibrational energy redistribution (IVR) on a subpicosecond (0.15–0.35 ps) time scale, and the 0.75–1 ns lifetime of the S_1_ (ππ*) state.^28,34^ In cyclohexane solution, the fluorescence lifetime of the chlorobenzene S_1_ state is similar, and the lifetime of the T_1_(ππ*) state was measured to be about 1 μs using phosphorescence and transient absorption spectroscopy.^25,35^ The shorter (∼80 ps) S_1_-state lifetime reported by Park et al. for chlorobenzene photoexcited at 267 nm in CCl_4_ solution^36^ could be because of excited-state electron-transfer reactions with the chlorinated solvent.

The principal UV-photoproducts of gas-phase chlorobenzene are a phenyl radical and a chlorine atom.^27,31^ In solution, the possibility that UV photodissociation of aryl halides instead makes aryl cations has been investigated previously, with a proposal that the aryl cations form by electron transfer between the radical pairs made by bond homolysis.^1,26,37−39^ However, the posited electron transfer has not been verified spectroscopically. The first ionization energy of a phenyl radical is >8 eV,^40^ and the electron affinity of a chlorine atom is 3.6 eV.^41,42^ Therefore, for the electron-transfer mechanism to be spontaneous, the ionic products must be strongly stabilized by a polar solvent and their mutual Coulomb attraction at short-range. Alternatively, pathways involving ion-pair states of the parent molecule might be responsible for heterolytic bond dissociation.

Here, we examine the proposition that phenyl cations form from UV-photoexcited chlorobenzene using ultrafast transient absorption spectroscopy to explore possible homolytic and heterolytic bond dissociation pathways. Following homolytic bond cleavage, phenyl radicals that escape geminate recombination can abstract a hydrogen atom from solvent molecules such as cyclohexane, ethanol (EtOH), or acetonitrile.^26^ Any phenyl cations formed from UV-excited PhCl will react with acetonitrile and other nucleophilic solvents or cosolutes.^20,21^ In contrast, perfluorinated organic solvents do not react with intermediates such as singlet and triplet carbenes^43,44^ and are expected to be unreactive toward phenyl cations. Therefore, we compare the chlorobenzene photochemical dynamics in perfluorohexane (PFH) with its photochemistry in common organic solvents. Cyclohexane and PFH serve as nonpolar solvent environments, whereas EtOH and 2,2,2-trifluoroethanol are chosen as representative polar and protic solvents. Moreover, fluorocarbons like PFH can dissolve significantly larger amounts of O_2_ or N_2_ than most common solvents,^45^ which could facilitate nitrogen capture by phenyl cations. Chlorinated solvents are avoided because of possible electron-transfer reactions with PhCl (S_1_) and because they show their own photochemistry when subjected to ultrafast UV laser pulses.^36,46−49^

## 2 Experimental and Computational Methods

Chlorobenzene (PhCl, Acros Organics, 99.9% for HPLC) was used as received and was dissolved in cyclohexane (Acros Organics, 99+% for spectroscopy), perfluorohexane (Aldrich, 99%), EtOH (Aldrich, for HPLC, ≥99.8%), or 2,2,2-trifluoroethanol (Acros, 99.8%) using an ultrasonic bath to make 0.2 M solutions. Transient electronic absorption spectroscopy (TEAS) measurements used an ultrafast laser system described in previous publications,^50,51^ and featuring a white-light continuum (WLC) probe spanning 340–700 nm. Solutions were continuously circulated through a Harrick cell fitted with CaF_2_ windows separated by 200 μm-thick polytetrafluoroethylene (PTFE) spacers. The same Harrick cells were also used for steady-state UV–vis absorption spectroscopy with a GENESYS 10S UV–vis spectrophotometer (Thermo Scientific). Steady-state IR absorption spectra were measured by a Spectrum Two FTIR spectrometer (PerkinElmer), using a Harrick cell with 100 μm PTFE spacers. Transient vibrational absorption spectroscopy (TVAS) measurements reported in Supporting Information were made with the LIFEtime laser facility located at the STFC Rutherford Appleton Laboratory, which is described in detail elsewhere.^52^

DFT calculations using the CAM-B3LYP/aug-cc-pVTZ level of theory obtained energies and vibrational frequencies of the ground state species,^53−57^ and the TD-DFT CAM-B3LYP/aug-cc-pVTZ method was used to compute the optimized structures and vibrational frequencies for electronically excited states.^58,59^ These choices of functionals and basis sets followed the methods used to compute charge-transfer complexes of carbon tetrachloride.^46^ Natural hybrid bond orbital (NBO) and natural population analysis (NPA) were implemented to understand charge distributions and electron localizations.^60−63^ All the calculations were performed using the Gaussian 09 package.^64^

## 3 Results and Discussion

### 3.1 Transient Electronic Absorption Spectra for UV-Photoexcited PhCl in Nonpolar Solvents

The steady-state UV absorption spectra of the longest wavelength PhCl bands are compared in Figure S1 for cyclohexane and PFH solutions. Vibrational structures are resolved in these electronic absorption spectra because of the nanosecond lifetime of the S_1_ (ππ*) state. The excitation wavelengths for TEAS measurements were chosen as 245 or 270 nm to explore the effects of direct excitation to the S_1_ (ππ*) state with different amounts of internal energy. With 245 nm excitation, the crossing point from the S_1_ (ππ*) state to the repulsive T_5_ (nσ*) state should be energetically accessible, whereas at 270 nm the main photodissociation pathway is expected to be via the vibrationally hot ground state.^29^

**Figure 1:**
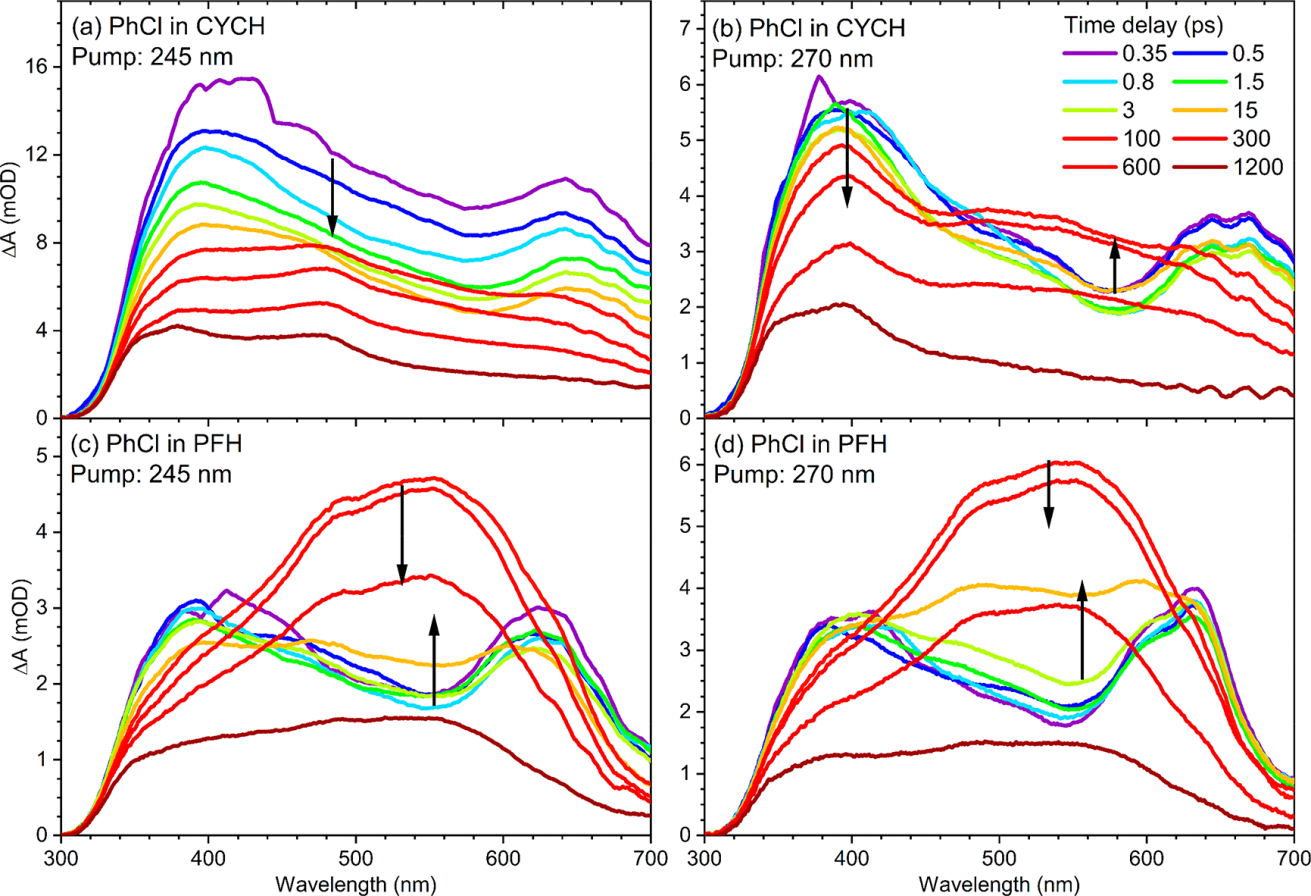
Transient electronic absorption spectra of chlorobenzene in (a) cyclohexane with excitation at 245 nm, (b) cyclohexane with excitation at 270 nm, (c) PFH with excitation at 245 nm, and (d) PFH with excitation at 270 nm. Spectra recorded at different time delays are indicated by colored lines, with the color key provided in panel (b). Black arrows indicate the directions of change in intensity of transient features. Loss of intensity for transient absorption features at wavelengths below 350 nm is caused by the short-wavelength cutoff in the WLC probe used for the measurements.

Example transient spectra of PhCl in cyclohexane and PFH obtained with pump laser wavelengths of 245 and 270 nm are shown in Figure 1. The most noticeable difference between the TEAS data in the two solvents is the prominence of the band centered near 540 nm for PhCl in PFH. This band rises at time delays around 100 ps and then decays. With changes to the pump laser wavelength, the early time (Δ*t* = 0.35 ps) TEA spectra do not change dramatically, but some differences are apparent between the TEA spectra obtained in cyclohexane following excitation at wavelengths of 245 and 270 nm. The rising feature at around 540 nm is more apparent for 270 nm than for 245 nm excitation, and the late time (Δ*t* = 1200 ps) TEAS measurements in cyclohexane solutions excited at 270 nm (Figure 1b) show a peak at 400 nm. In contrast, a pair of peaks are observed at 375 and 480 nm in the late time spectrum for PhCl in cyclohexane excited at 245 nm (Figure 1a). These differences suggest some changes to the photoproducts at the two excitation wavelengths for chlorobenzene solutions in cyclohexane. Although these products cannot be definitively identified from our TEAS measurements, a proposed assignment is discussed below. Little, if any, pump wavelength dependence to the TEAS measurements is evident for PFH solutions of chlorobenzene.

The TEA spectra were decomposed into constituent bands using the KOALA software package to extract kinetic information.^65^ Examples of this spectral decomposition are shown in Figure S2 of the Supporting Information. The analysis used three basis functions for chlorobenzene TEA spectra obtained in each of the two solvents and for each of the two separate excitation wavelengths. The first basis function was chosen to be the TEA spectrum at an early delay time, when the S_1_ state is populated and has not evolved to different states. The best-fit magnitude of this contribution to the overall spectrum at any time delay represents the S_1_ state population. We see no clear-cut evidence for homolytic bond cleavage on this short time scale. The second basis function was chosen to capture the contribution from the absorption band around 540 nm and was obtained as the difference between transient spectra measured at mid- and early time delays, the latter scaled to account for loss of S_1_ absorption. It fits the feature observed to rise for delays around 100 ps, which becomes prominent in the midtime spectra. Finally, a late-time (>1 ns) spectrum was chosen for each set of experimental conditions to describe absorption by photoproducts.

**Figure 2:**
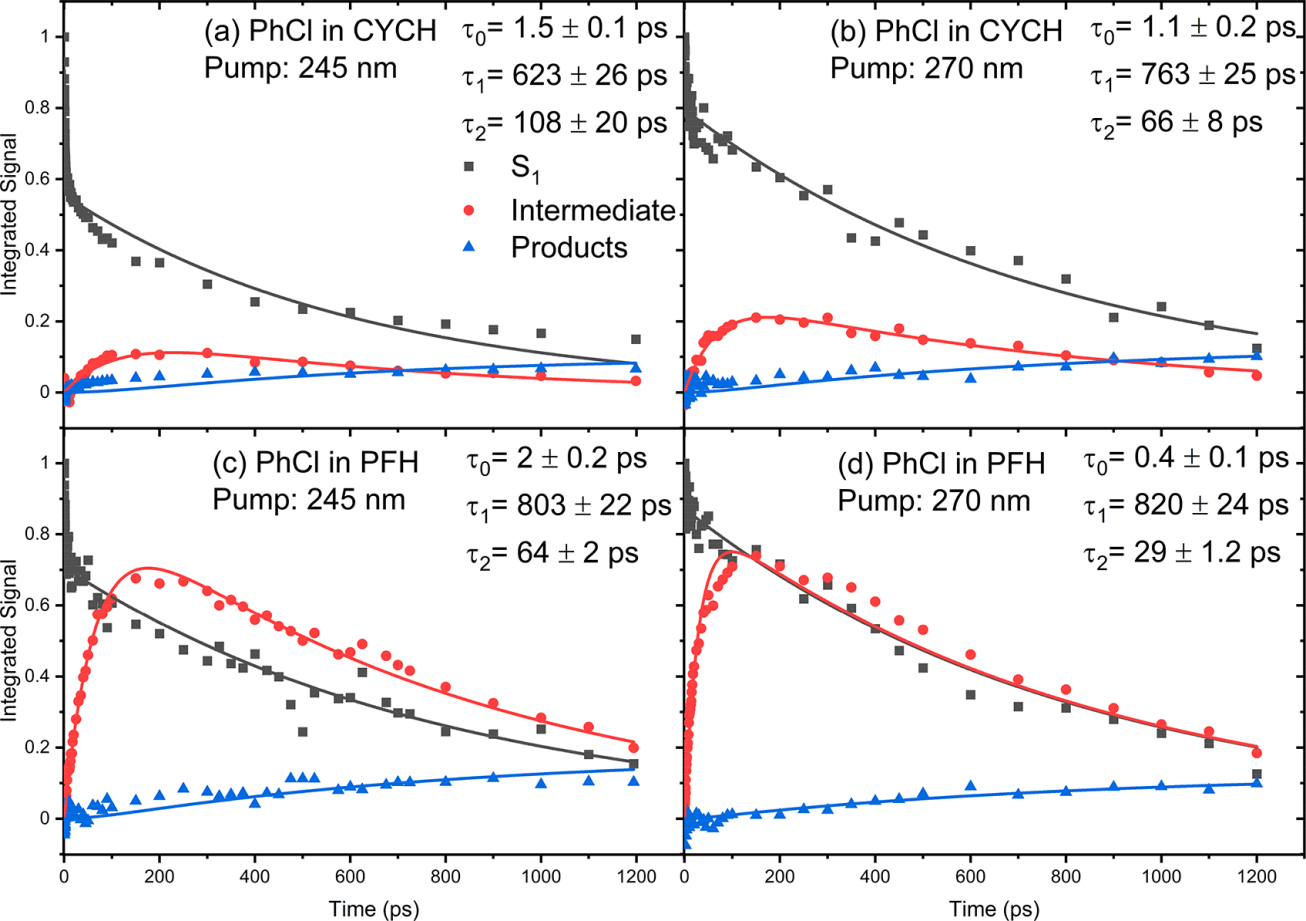
Photochemical kinetics derived from spectral decomposition of TEA spectra for chlorobenzene in (a) cyclohexane with 245 nm excitation; (b) cyclohexane with 270 nm excitation; (c) PFH with 245 nm excitation; and (d) PFH with 270 nm excitation. Within each panel, the kinetic traces are globally fitted (solid lines) with exponential functions modeling sequential reaction kinetics (see main text) to extract the time constants listed. Black symbols and lines represent the intensity of the PhCl (S_1_) excited-state absorption, whereas red and blue symbols and lines show the time-dependent band intensities for intermediates and photoproducts, respectively.

The integrated intensities associated with the three fitted basis functions, normalized to a maximum value of 1.0, are plotted as a function of time in Figure 2 for the two solutions and both excitation wavelengths. The figure panels also show best global fits to a consecutive reaction model and the resulting time constants. The kinetic model used is PhCl (S_1_) → I → P, where I and P denote an intermediate and the photoproducts, respectively. The decay of the S_1_-state absorption is biexponential, so an additional time constant was added to the fit function for the S_1_ state population. The first of the S_1_ decay time constants (τ_0_) has a value around 1 ps, and it is attributed to IVR in the S_1_ state, perhaps also in conjunction with vibrational energy transfer to the solvent. This component is more prominent for the shorter wavelength UV excitation, consistent with a greater initial internal energy in the photoexcited PhCl (S_1_) molecules. Prompt C–Cl bond dissociation is considered unlikely because the S_1_ state is bound, and the time constant appears to be too small for ISC to the T_5_ (nσ*) repulsive state, although crossing to the dissociative S_4_ (nσ*) state^29^ may play a role if it is energetically accessible at the shorter excitation wavelength. The second time constant (τ_1_) of around 600–800 ps accounts for the lifetime of the S_1_ state and is similar to values (∼750 ps to 1 ns) reported previously for gas-phase PhCl photoexcited at a wavelength of 270 nm^28,34^ as well as fluorescence lifetime measurements of 740 ± 90 ps in cyclohexane, with concentrations from 5 mM to 40 mM,^35^ and 786 ± 12 ps in acetonitrile solutions.^66^ The insensitivity of the lifetime of the S_1_ state to the concentration of PhCl argues against bimolecular relaxation mechanisms in which the PhCl (S_1_) is quenched by reaction, energy transfer, or electron transfer with another PhCl (S_0_) molecule. The τ_1_ value is slightly smaller following 245 nm excitation in cyclohexane, which could be a consequence of some photoexcited molecules crossing to a repulsive state.^29^ However, this pattern is not seen for the corresponding measurements in PFH; instead, the S_1_ state lifetime does not change significantly for the two excitation wavelengths.

The kinetics of the intermediate absorption band show a rise and a decay. At each excitation wavelength and for each solvent, the decay of the S_1_ population, the growth and decay of the intermediate, and the rise in product absorption can all be accounted for by the same two values for the time constants τ_1_ and τ_2_, as shown in Figure 2. Therefore, the time constant for the intermediate formation was globally fitted to the same τ_1_ value as was obtained from the S_1_ population decay, and the concurrently fitted τ_2_ parameters correspond to the lifetimes of this intermediate species in different solvents. The derived τ_2_ values of 30–110 ps show that the loss of the intermediate is faster than its formation. Hence, the concentration of the intermediate remains low, so its observation requires an absorption band with a large oscillator strength.

The late-time basis function used for the spectral decomposition could represent products of the photochemistry such as phenyl radicals, or alternatively a long-lived triplet state of PhCl. However, the known and weak (with an extinction coefficient, ε = 2.8 M^–1^ cm^–1^), structured electronic absorption band of the phenyl radical, extending from ∼400 nm up to 510 nm in an Ar matrix,^67^ is not a good match to the observed photoproduct absorption. Instead, phenyl radicals could be reacting with oxygen dissolved in the solution to form a phenylperoxyl radical absorbing at ∼460 nm.^26^ Excited-state absorption (ESA) from PhCl (T_1_) has been argued previously to appear at wavelengths around 300 nm, and a long-wavelength shoulder of this T_1_ state absorption band may extend into our probe window.^25,26^ PhCl^+^, with a computed absorption band at 410 nm (see Figure S3 of Supporting Information), could also account for the observed late-time band, but it is unclear how this species might form in our experiments other than by direct ionization by the excitation laser pulses. Absorption of more than one UV photon would be required to ionize PhCl, which has a gas-phase ionization energy >9 eV.^68^ A posited assignment of some of the late-time absorption to *ortho*-benzyne photoproducts following HCl elimination is discussed later.

The focus here is on the intermediate species responsible for the absorption band that first rises and then decays in our measurement time window. The identity of this intermediate remains to be determined, but some candidates can be discounted, and alternatives consistent with the available experimental data are proposed. The mismatch between the strength and wavelength range of the observed transient absorption feature and the known spectrum of the phenyl radical^67^ argues against this candidate assignment. The amplitude of the contribution from the intermediate basis spectrum is larger for PhCl photoexcited in PFH than in cyclohexane. Hence, the prominence of the intermediate species responsible may relate to specific properties of PFH, namely high gas solubility and chemical inertness. Perfluorohexane can dissolve larger amounts of gaseous N_2_ and O_2_ than cyclohexane, so the intermediate band might arise from compounds containing nitrogen or oxygen such as a diazonium cation or phenylperoxyl radical. However, those compounds do not absorb at wavelengths around 560 nm where the intermediate absorption is greatest.^21,22,26^ Because of the inertness of PFH, any radical pairs formed by homolytic bond cleavage will not react with the solvent, whereas cyclohexane can undergo hydrogen abstraction by a phenyl radical or a chlorine atom to make a cyclohexyl radical and either benzene or HCl.^26^ Although the longer lifetimes of such radical pairs in PFH may permit the previously proposed electron transfer to form a phenyl cation and chloride anion (i.e., an ion pair), the low polarity of PFH, with a dielectric constant of ∼1.7, argues against this charge-transfer pathway because the resulting ions will not be favorably solvated.^69^

A charge-transfer-to-solvent (CTTS) band of Cl^–^ is a further candidate assignment for the intermediate absorption feature, but is observed at shorter wavelengths in aqueous solution.^70^ It is also expected to be outside our observation window for Figure 1 in the weakly interacting PFH solvent because the gas-phase electron affinity of Cl^–^ is 3.61 eV.^71^ Computational predictions of electronic transitions argue against assignment of the intermediate absorption to the phenyl cation. This cation has singlet and triplet spin states, with the singlet phenyl cation being more stable than its triplet counterpart. CASPT2/cc-pVDZ calculations predict that the singlet phenyl cation absorbs at 186 nm,^72^ which is beyond our measurement window. The similar geometries of the triplet phenyl cation and phenyl radical suggest the former might be a short-lived intermediate between the phenyl radical and formation of a singlet phenyl cation, although the ionization and ISC dynamics have not been simulated.^17^ At UV wavelengths of 248 nm or less, the bond dissociation in photoexcited, gas-phase PhCl can occur through a triplet state with nσ* character,^29^ hence the radical pair from homolytic bond cleavage for the corresponding process in solution will initially form with correlated electron spins of overall triplet character. Prompt electron transfer would then favor formation of Ph^+^ in its triplet state (because the partner Cl^–^ is a singlet spin anion). Nevertheless, our calculations (Figure S4 in the Supporting Information) suggest any electronic absorption bands of the triplet phenyl cation are at wavelengths below 400 nm and are weak.

As an alternative to an ion pair comprising two separated ions, the intermediate absorption may instead come from a charge-transfer band of a complex with ion-pair character, denoted here as Ph^+^-Cl^–^. Similar complexes with ion-pair character can be formed by photoexcitation of halogenated alkanes and have been identified as reactive intermediates in both experimental and theoretical studies.^73−77^ The corresponding complexes of CCl_4_ and CHCl_3_, which are referred to in the photochemistry and pulsed radiolysis literature as *iso-*CCl_4_ and *iso-*CHCl_3_ to indicate their isomeric structures, exhibit strong ion pair character. For example, computational evidence shows that *iso*-CCl_4_ can be regarded as a Cl^–^-ClCCl_2_^+^ species.^46^ These *iso* species have strong and broad absorption bands around 500 nm that closely resemble the intermediate features seen here for PhCl.^46,78^ One plausible candidate assignment for the intermediates observed in our TEAS measurements is therefore an *iso*-PhCl complex with charge-transfer character. In addition to the similarity of these distinctive spectral signatures, further evidence for the formation of such charge-transfer complexes from UV-photoexcited PhCl requires computational investigation of the energetics of such an *iso*-PhCl complex and theoretical prediction of its absorption spectrum. The outcomes of our computational studies are reported in Section 3.2.

### 3.2 Computational Investigation of Ph^+^-Cl^–^ Complexes

**Scheme 1:**
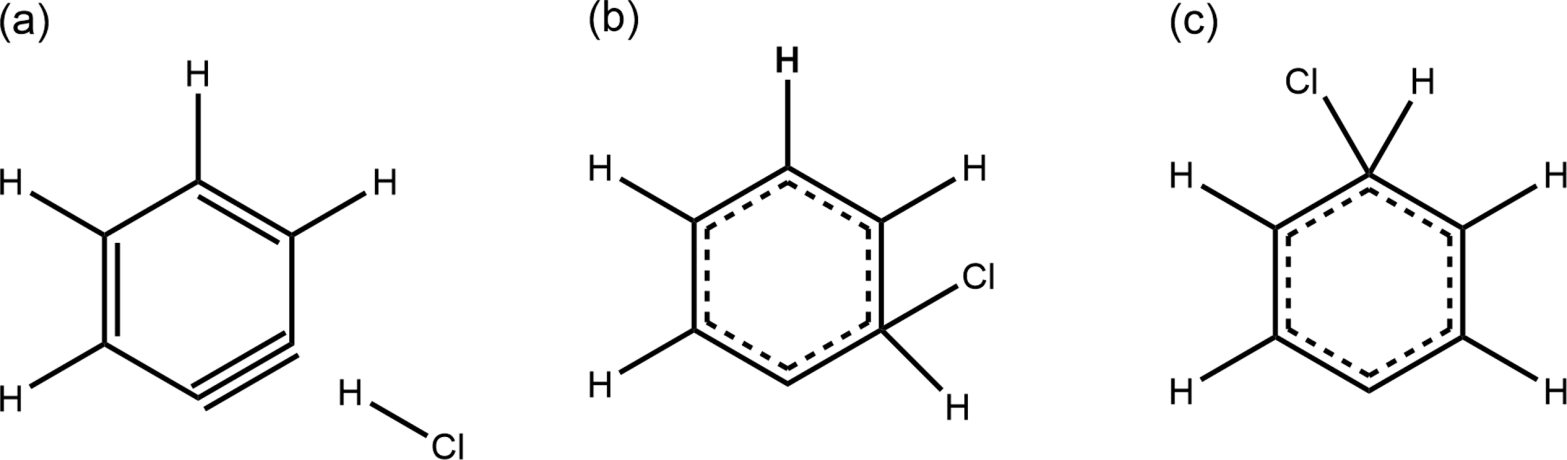
Schematic Skeleton Structures of *iso*-PhCl Complexes in (a) Singlet *ortho*, (b) Triplet *ortho*, and (c) Singlet and Triplet *para* Forms The computed optimized geometries of the *iso*-PhCl complexes on which these structures are based are shown in Figure S6.

To guide our identification of potential structures for Ph^+^-Cl^–^, the resonance forms of the phenyl cation shown in Figure S5 indicate which carbon atoms carry greater partial positive charges and, hence, are more likely to bind the partner chloride ion. This analysis suggests the *ortho* and the *para* sites as the starting points for calculations to explore where the chloride ion might attach to make an *iso*-PhCl species. The resulting complex will have both ion-pair and carbene character, making it sufficiently reactive with cyclohexane to be difficult to observe in our TEAS measurements, whereas carbenes do not react with perfluorocarbon solvents.^44,79^ Analogous to the electronic structures of carbenes,^80^ the proposed *iso*-PhCl structures are expected to have energetically low-lying singlet and triplet states. Therefore, the optimized structures of singlet and triplet *iso*-PhCl complexes were calculated using quantum chemistry methods and are reported in Figure S6, together with the atom numbering scheme used here. Scheme 1 shows schematic representations of these computed structures.

The singlet *orthoiso*-PhCl complex shown schematically in Scheme 1a appears to be associated with H–Cl elimination because the Cl atom/anion does not bond with a carbon atom of the phenyl radical/cation. Instead, the optimized geometries favor separated *ortho*-benzyne and HCl. In all the other computed complexes, the Cl is attached to a carbon, as shown in Scheme 1b,c. A natural population analysis was performed to identify whether the bonds in these *iso*-PhCl complexes have ion-pair character, with the results reported in Table S1.^60−63^ This table also summarizes the computed energies of the *iso*-PhCl complexes relative to ground-state PhCl. The natural charges of the Cl atom in the triplet *iso*-PhCl complexes are significantly more negative than for the ground-state PhCl isomer. Similarly, the natural charge of carbon atom C1 (with carbene character) in the triplet *iso*-PhCl complexes is more positive than for the ground-state PhCl isomer. These outcomes confirm the pronounced ion-pair character of the triplet complexes.

Figure S7 shows the calculated electronic absorption spectra of the *iso*-PhCl complexes. For the triplet-spin complexes, a strong absorption band is predicted at wavelengths around 400 nm. In contrast, the oscillator strengths for absorption bands of the singlet complexes falling within our TEAS observation window are smaller, with the stronger transitions predicted to lie at wavelengths below 300 nm. As a result, the intermediate band observed in our TEA spectra is attributed to the triplet *iso*-PhCl complexes with ion-pair character, consistent with photochemical dynamics via a dissociative triplet excited state of PhCl such as the ^3^nσ* state. The corresponding singlet complexes may be formed either directly or via the triplet complexes, but they would not be seen in our TEAS measurement window. Although there is a wavelength mismatch between the experimentally observed intermediate bands and the computed transitions of the triplet *iso*-PhCl complexes, a similar level of disagreement between experiment and calculations was reported previously by Abou-Chahine et al. for the corresponding *iso-*CCl_4_ and *iso*-CHCl_3_ bands.^46^ When it comes to the feasibility of formation of the various *iso*-PhCl structures, the calculated energies (Table S1) suggest that they are energetically accessible with excitation wavelengths of 270 nm or shorter, corresponding to photon energies of 4.6 eV or above.

The energy of the singlet *ortho* complex is the lowest among all the *iso*-PhCl species considered. The observed decay of the band assigned here to triplet *iso*-PhCl complexes, with time constants of 30–110 ps (Figure 2), may therefore be because of ISC to the lower-lying singlet form, which separates into *ortho*-benzyne and HCl (Scheme 1a). This proposed relaxation mechanism is supported by comparison of the product band observed in the late time TEA spectra in Figure 1 (and the product basis function in Figure S2 in the Supporting Information) with previous reports of *ortho*-benzyne spectra. In an Ar matrix, *ortho*-benzyne has an electronic absorption band at ∼377 nm,^81^ and previous transient absorption experiments reported a 370 nm band assigned to an intermediate containing an *ortho*-benzyne moiety.^82^ A similar structure to those considered here for the *iso*-PhCl complexes was recently computed by Behera et al., who suggested its involvement in HCl elimination from the 266 nm photolysis of gas-phase PhCl.^83^ They observed ro-vibrational spectral lines assigned not only to HCl but also to acetylene (C_2_H_2_) and 1,3-butadiyne (C_4_H_2_) which may arise from further degradation of internally excited *ortho*-benzyne.^83^

Transient vibrational absorption spectra obtained for the same two solutions of PhCl, but using a UV excitation wavelength of 270 nm and a mid-IR probe pulse spanning 1470–1630 cm^–1^, are shown in Figure S8 of the Supporting Information. These measurements extend to longer delay times than was possible with our TEAS setup. Spectral analysis and computational predictions of vibrational frequencies both support the assignment of the observed intermediate species to the triplet-spin *iso-*PhCl complexes, as discussed in the Supporting Information. No spectral signatures of long-lived photoproducts are identified in our mid-IR spectral window, but the kinetics of the observed transient absorption bands can be fitted with the same time constants derived from the TEAS measurements (Figure S9). Moreover, ground-state bleach features, corresponding to depletion of PhCl (S_0_) mid-IR bands by UV-photoexcitation, do not recover within 5 ns, suggesting the photoinduced dynamics do not repopulate vibrationally relaxed PhCl in the S_0_ state. Hence, no significant geminate recombination of Ph + Cl radicals, or relaxation of *iso*-PhCl back to PhCl, occurs within our measurement time window.

### 3.3 TEA Spectra of UV-Photoexcited PhCl in Polar Solvents

**Figure 3:**
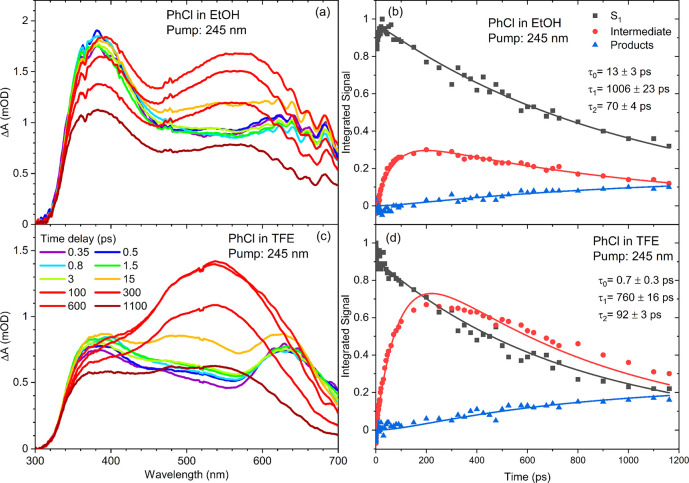
TEA spectra of 245 nm photoexcited PhCl in (a) EtOH and (c) TFE. The WLC used for spectroscopic detection rapidly loses intensity at wavelengths below 350 nm. The kinetics derived from spectral decomposition are shown in (b) for EtOH and (d) for TFE solutions. The kinetic data points (solid symbols) are globally fitted (solid lines) with exponential functions modeling sequential reaction kinetics to extract the time constants listed. Black symbols and lines represent the intensity of the PhCl (S_1_) excited-state absorption, whereas red and blue symbols and lines show the time-dependent band intensities for intermediates and photoproducts, respectively.

The triplet *iso*-PhCl complexes described above have pronounced ion-pair character, hence they should be stabilized in a polar solvent environment. To test this idea, TEA spectra of UV- photoexcited PhCl were measured in EtOH and trifluoroethanol (TFE), with the results shown in Figure 3. The TEA spectra in these polar solvents reveal similar features to the ones observed in the nonpolar cyclohexane and PFH solvents, and these were analyzed using the same approaches to spectral decomposition and kinetic fitting. The intermediate band is more obvious for 245 nm photoexcited PhCl in EtOH than in cyclohexane (cf., Figure 1a), but it is not as pronounced as in the fluorinated solvents PFH and TFE, indicating preferential formation of the ion-pair isomer in more fluorinated solvents. The τ_2_ time constant assigned to decay of the intermediate band is smaller in EtOH than in TFE, which may reflect more rapid ISC to the lower-lying singlet state or greater reactivity of triplet *iso*-PhCl with the former solvent. The larger τ_2_ time constant for this intermediate decay in TFE than in PFH could indicate stabilization of the charge-transfer character in the more polar solvent. Hence, the TEA spectra obtained in different solvent environments appear consistent with our assignment of an intermediate species corresponding to a PhCl isomer with triplet spin and both ion-pair and carbene character.

## 4 Summary and Conclusions

The photochemistry of S_1_ (^1^ππ*)-state PhCl has been studied at different UV excitation wavelengths and in four solvents chosen for their contrasting polarities and likely reactivities. The TEA spectra of PhCl in cyclohexane show an apparent excitation wavelength dependence from 245 to 270 nm, but this is less evident in PFH. Although previous gas-phase studies of PhCl photochemistry suggest there are accessible crossings to repulsive states of nσ* character when the UV excitation wavelength is 248 nm or shorter, in solution, the photoexcited molecules will undergo vibrational cooling in competition with IC or ISC to these dissociative states. The lifetime of the S_1_ state approaches 1 ns, yet most of the population in the S_1_ state does not return to the ground state because the ground-state bleach features observed by TVAS do not recover significantly on this time scale. The reaction pathways of photoexcited PhCl (S_1_, ^1^ππ*) were previously suggested to be homolytic bond cleavage to Ph + Cl radicals,^29,30^ HCl elimination,^83^ and ISC to the T_1_ state.^84^ Here, we propose a solvent-sensitive reaction pathway that involves the formation of intermediates argued to be isomers of PhCl (denoted as *iso*-PhCl) with pronounced charge-transfer character. These isomeric forms are revealed by their strong electronic absorption bands in the visible region, the intensities of which vary in different solvent environments.

Our calculations indicate that two *iso*-PhCl complexes could be assigned as the intermediates, namely a triplet *ortho* complex (Scheme 1b) and a triplet *para* complex (Scheme 1c). Using the NPA method, these complexes are shown to have charge-separated character in which the chlorine atom carries more negative charge than for PhCl in its electronic ground state. Comparison of the *iso*-PhCl yields in cyclohexane (Figure 1a) and EtOH (Figure 3a) suggests they depend on the polarity of the solvent, consistent with this charge-separated character. Lazzaroni et al. used σ-nucleophiles and π-nucleophiles intended to trap singlet and triplet phenyl cations posited to form from the UV photodissociation of PhCl in various solvents.^25^ We propose that this trapping may instead be of the phenyl-cation constituent of an *iso*-PhCl complex (with carbene character) rather than free Ph^+^. The prior results showed the yield of trapped phenyl cations is higher in TFE (p*K*_a_ = 12.37) than in methanol (p*K*_a_ = 15.49), from which Lazzaroni et al. suggested a dependence on the acidity of the solvents rather than the polarity.^25^ As our own observations in Figure 3 show, the *iso*-PhCl band in TFE is indeed stronger than in EtOH. Nevertheless, we also observe that this peak is most apparent in the nonacidic and nonpolar solvent PFH. Because the triplet *iso*-PhCl complexes are predicted to have reactive carbene character, we propose that their yields depend not only on the polarity but also the reactivity of the solvent toward carbenes.

Following the formation of *iso*-PhCl intermediates with ion-pair character, subsequent reaction pathways remain unclear because no product absorption peaks are observed in the probe window for our mid-IR TVA spectra, and the broad product bands seen by TEAS are not straightforwardly assigned. Evidence from the late-time TEAS measurements tentatively suggests that initially formed triplet *iso*-PhCl relaxes to the lower energy singlet *iso*-PhCl form and eliminates HCl to form *ortho*-benzyne (consistent with our calculated structures in Figure S6). In polar solvents, the solvent molecules may intervene between the Ph^+^ and Cl^–^ moieties in the *iso*-PhCl, resulting in separation of the ionic fragments. This proposition is supported by MRCI calculations of photoexcited chloromethane in which a solvent water molecule inserted between CH_3_^+^ and Cl^–^ fragments.^85^

Previous discussions of a role for phenyl cations in synthetic chemical pathways have suggested that they form via a triplet state of UV-photoexcited PhCl (most likely, the T_1_ state).^25^ The lifetime of PhCl (T_1_, ^3^ππ) was measured to be 1 μs using nanosecond resolution transient absorption spectroscopy,^25,26^ reflecting its nondissociative character, but relaxation pathways were not identified. This lifetime is too long to account for our observations of *iso*-PhCl formation on 600–800 ps time scales, which match more closely the lifetime of the PhCl (S_1_, ^1^ππ*) state. We propose instead that a dissociative ^3^nσ* triplet state may be involved in *iso*-PhCl formation via a crossing with the S_1_ state that is accessible at thermal internal energies. Alternatively, the *iso*-PhCl may form following IC to the S_0_ state, but this pathway must then be able to compete with solvent quenching of the excess vibrational energy. This latter mechanism does not account for the formation of the *iso*-PhCl in a triplet state (unless ISC can occur at extended C–Cl distances during or after bond homolysis to Ph• + Cl•), and it appears to be inconsistent with the modest amount of ground-state bleach recovery observed in our TVAS measurements.

The interesting question remains of whether molecules like N_2_ might be activated and incorporated into organic molecules using UV photolysis of PhCl. While this activation may be because the *iso*-PhCl intermediates that we observe can subsequently fragment to Cl^–^ and the highly reactive Ph^+^ cations, an alternative proposition is that the carbene character of the *iso*-PhCl (whether in its triplet or singlet forms) allows reaction with N_2_ to make diazo compounds. Alternatively, the *ortho*-benzyne produced by loss of HCl from singlet *iso*-PhCl may be a reactive intermediate in N_2_ activation. Saturation of PFH solutions with N_2_ could offer an opportunity to study such reactions using the types of transient absorption spectroscopy methods applied in the current study.

## Notes

Data are available at the University of Bristol data repository, data-bris, at https://data.bris.ac.uk/data/dataset/3ucy0js12f8uj2m3mm3ihuy2dy.

## Supporting Information Available

Steady-state UV–vis absorption spectra of PhCl solutions in cyclohexane and PFH; examples of decomposition of transient absorption spectra; computed electronic absorption spectra for various reactive intermediates; computationally optimized structures of *iso*-PhCl complexes and analysis of their charge distributions; transient IR spectra of PhCl solutions in cyclohexane and PFH, and their analysis (PDF)
