# Supplementary material for: Charge-Separated Reactive Intermediates from the UV Photodissociation of Chlorobenzene in Solution
Source: J Phys Chem A. 2022 Sep 23;126(39):6934–43. doi: 10.1021/acs.jpca.2c05327 (PMC9549464; doi:10.1021/acs.jpca.2c05327)
Supplement: Supplementary file 1 — jp2c05327_si_001.pdf [file jp2c05327_si_001.pdf]

Supporting Information for
Charge-Separated Reactive Intermediates from the UV Photodissociation of Chlorobenzene in Solution

Min-Hsien Kao and Andrew J. Orr-Ewing*

School of Chemistry, University of Bristol, Cantock's Close, Bristol BS8 1TS, UK

* Author for correspondence: a.orr-ewing@bristol.ac.uk

Table of Figures

Figure S1. The UV absorption spectra of chlorobenzene 2

Figure S2. Examples of spectral decomposition of TEA spectra of chlorobenzene 3

Figure S3. Predicted electronic transitions of PhCl$^+$ 4

Figure S4. Predicted electronic transition of the triplet-spin phenyl cation.........................5

Figure S5. Resonance structures of the phenyl cation 5

Figure S6. Calculated optimized structures of iso-PhCl complexes.....................................6

Table S1. Natural charges of the atoms in ground state PhCl and in iso-PhCl complexes.....7

Figure S7. Calculated electronic absorption spectra of iso-PhCl complexes 8

Figure S8. TVA spectra of 270-nm UV-photoexcited PhCl 9

Figure S9. The kinetics of TVAS bands...10

Figure S10. The scaled calculated IR spectra 12

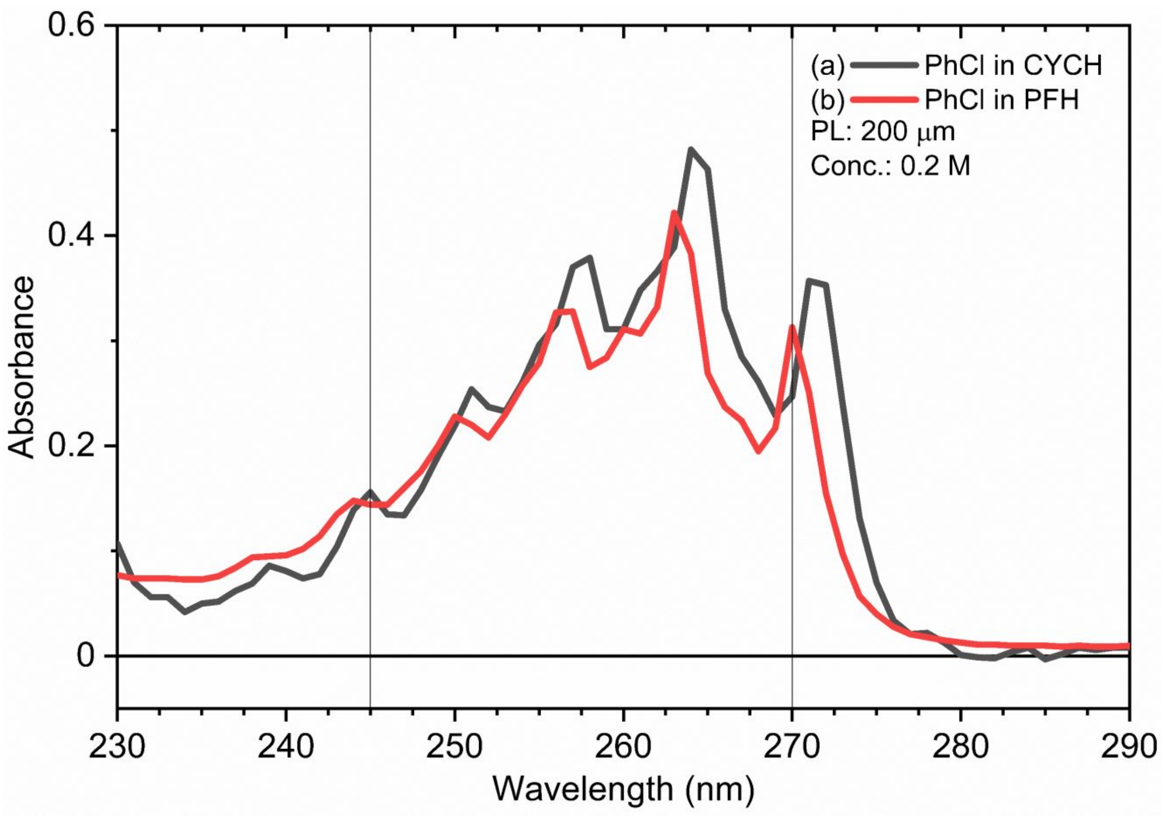

Figure S1. The 230 – 290 nm portions of the UV absorption spectra of chlorobenzene in (a) cyclohexane (CYCH) and (b) perfluorohexane (PFH). The sample pathlengths (PL) were 200 μm, and the concentrations were 0.2 M. Vertical lines show the UV wavelengths chosen for TEAS measurements.

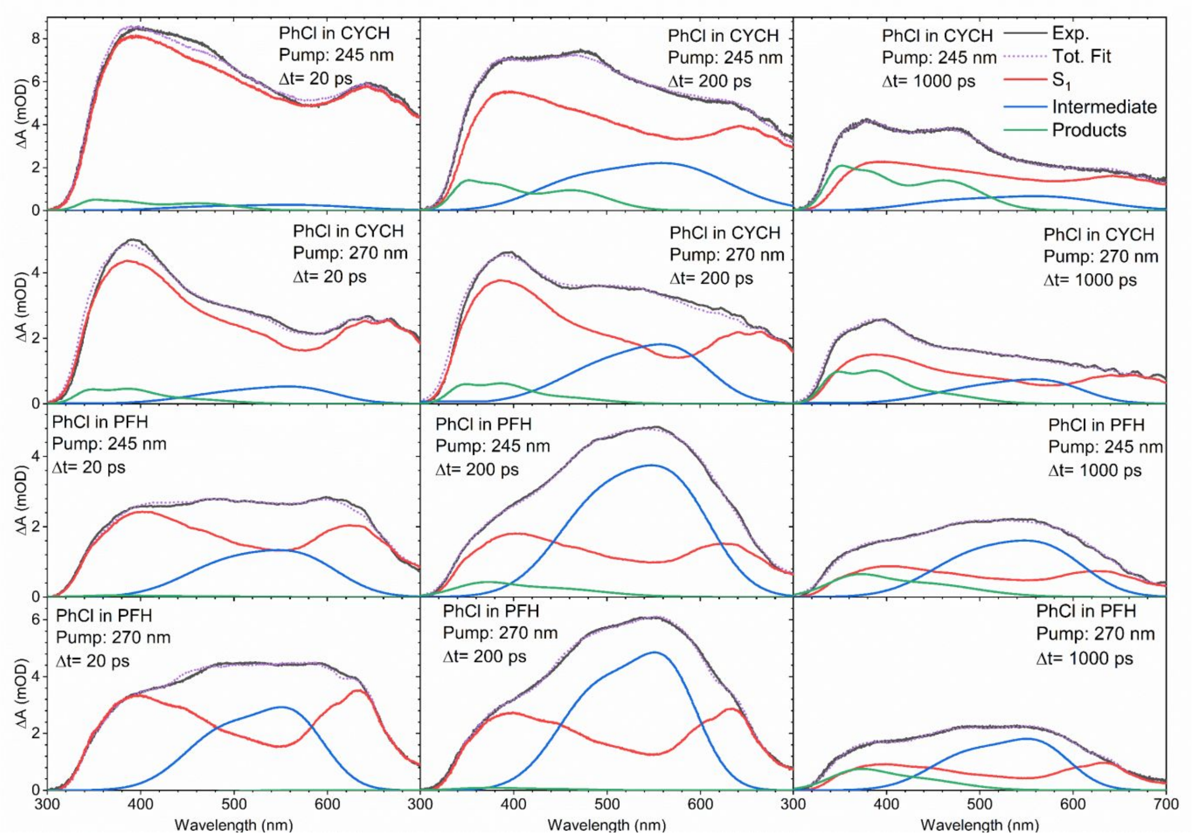

Figure S2. Examples of spectral decomposition of chlorobenzene TEA spectra for the solvents, time delays (Δt) and excitation wavelengths shown in each panel. This analysis was performed using the KOALA program.[1] in each panel, the grey solid line is an experimental spectrum, and the purple dotted line is the total fit spectrum that is the sum of contributions from three basis functions attributed to absorption from the PhCl S_1 state (red), an intermediate species (blue) and the photoproducts (green).

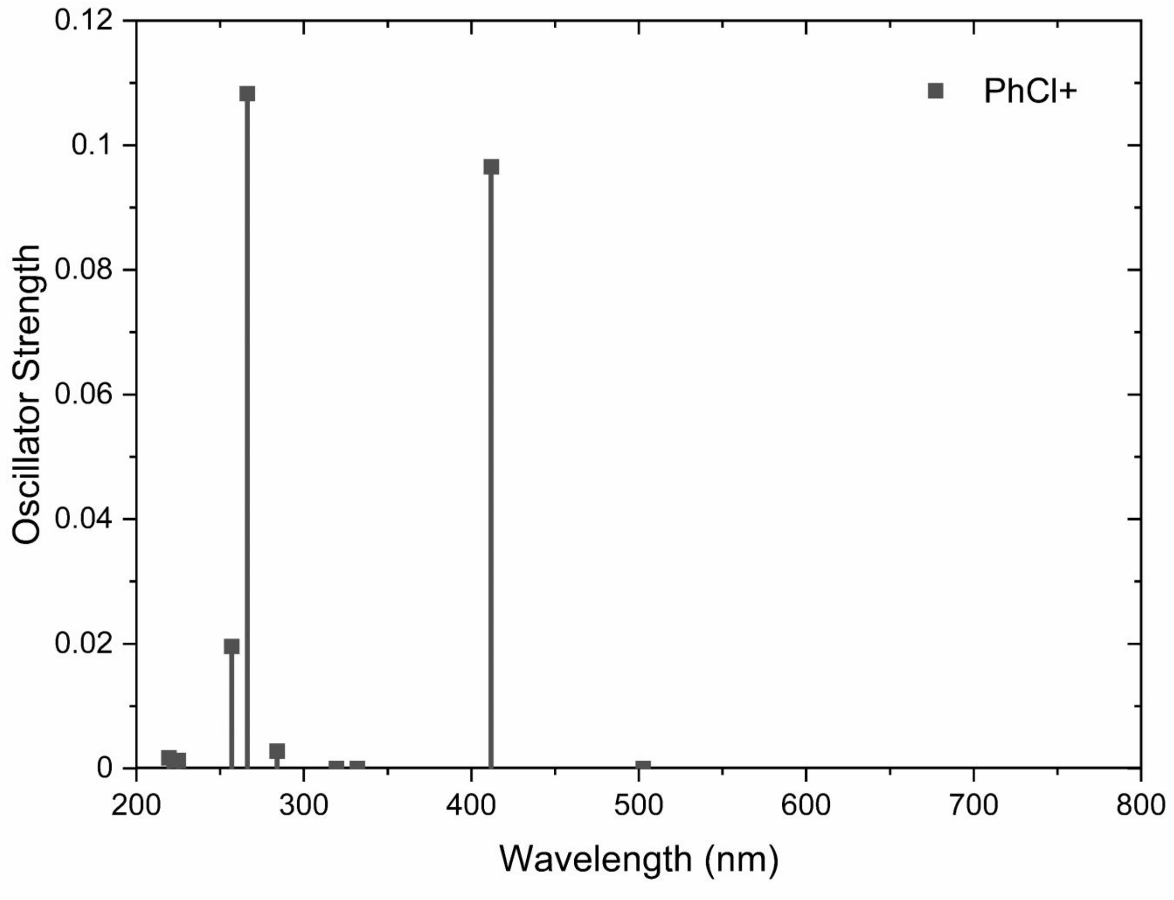

Figure S3. Predicted electronic transitions of $PhCl^{+}$ calculated at the TD-DFT CAM-B3LYP/aug-cc-pVTZ level of theory.

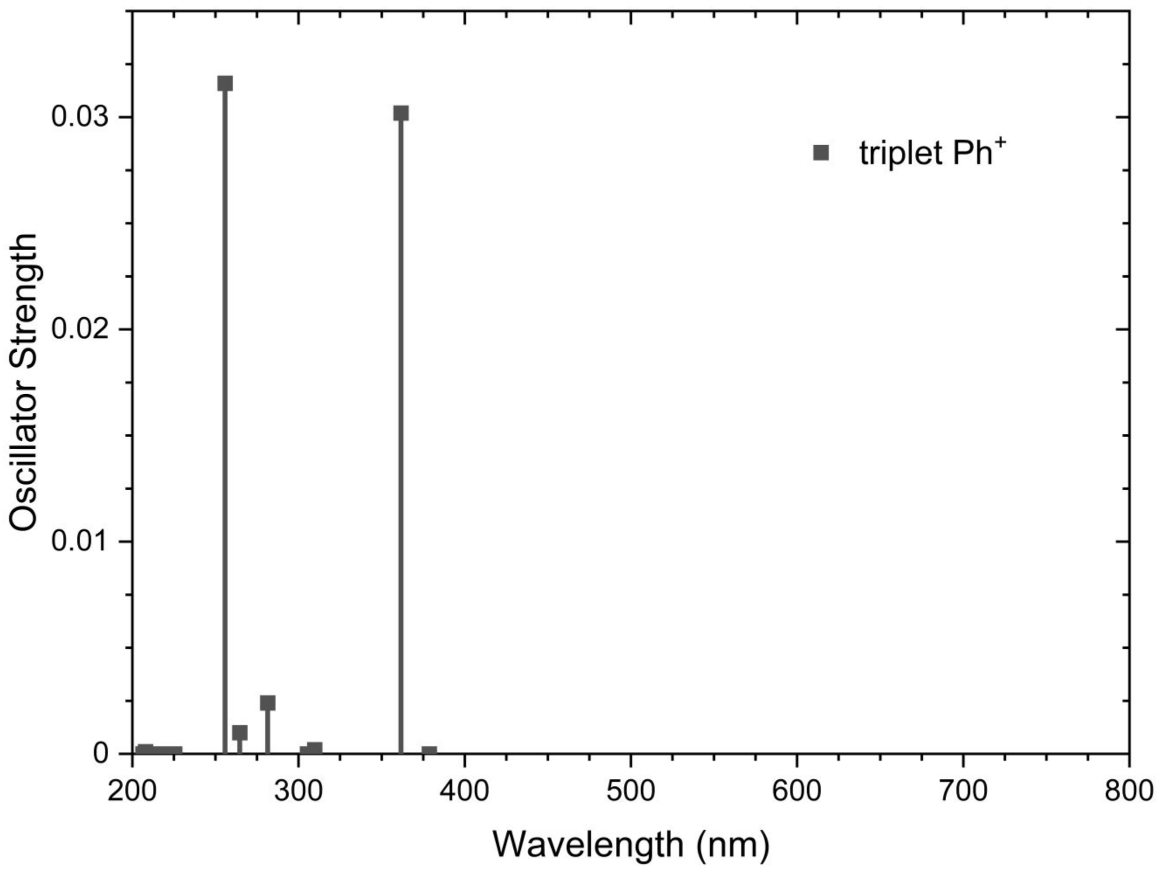

Figure S4. Predicted electronic transition of the triplet-spin phenyl cation (Ph^+) calculated at the TD-DFT CAM-B3LYP/aug-cc-pVTZ level of theory.

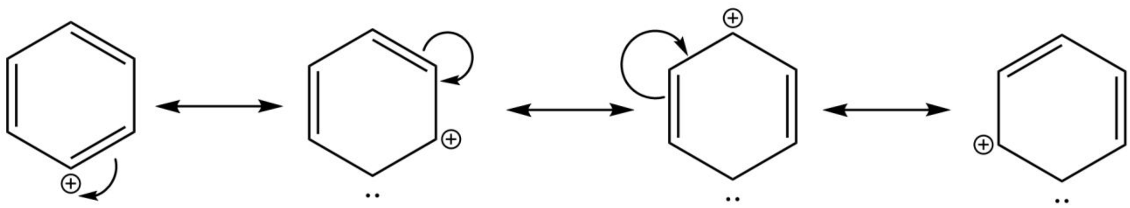

Figure S5. Resonance structures of the phenyl cation.

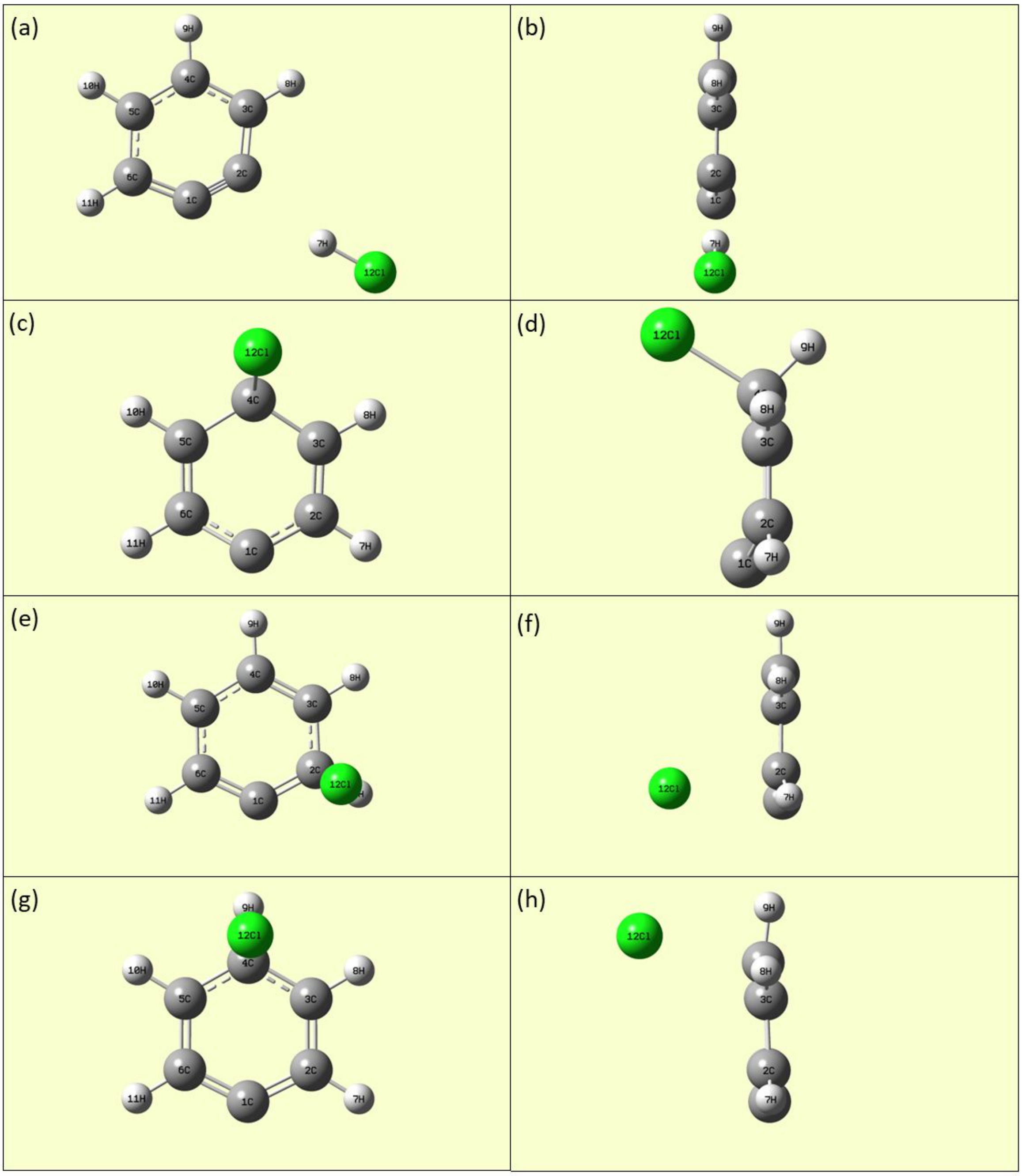

Figure S6. Calculated optimized structures of *iso*-PhCl complexes, shown from above and side-on perspectives: (a) and (b) the singlet ortho complex; (c) and (d) the singlet para complex; (e) and (f) the triplet ortho complex; (g) and (h) the triplet para complex. The atoms shown are carbon (grey), hydrogen (white) and chlorine (green). Calculations were performed at the TD-DFT CAM-B3LYP/aug-cc-pVTZ level of theory.

		ground-state PhCl	singlet ortho *iso*-PhCl	singlet para *iso*-PhCl	triplet ortho *iso*-PhCl	triplet para *iso*-PhCl
Atom	No	Natural Charge	Natural Charge	Natural Charge	Natural Charge	Natural Charge
C	1	-0.23	0.09	0.08	0.24	0.24
C	2	-0.19	-0.08	-0.32	-0.29	-0.33
C	3	-0.21	-0.22	-0.12	-0.15	-0.14
C	4	-0.19	-0.21	-0.24	-0.23	-0.18
C	5	-0.23	-0.20	-0.12	-0.13	-0.14
C	6	-0.02	-0.26	-0.32	-0.34	-0.33
H	7	0.22	0.27	0.22	0.25	0.23
H	8	0.21	0.22	0.22	0.22	0.22
H	9	0.21	0.22	0.25	0.22	0.24
H	10	0.21	0.22	0.22	0.21	0.22
H	11	0.22	0.23	0.22	0.23	0.23
Cl	12	-0.01	-0.28	-0.08	-0.25	-0.27
C-Cl bond length (Å)		1.74	H-Cl	1.84	2.46	2.45
Energy (eV)		0.00	3.53	4.22	3.72	3.68

Table S1. Natural charges of the atoms in ground state PhCl (S_0) and the singlet and triplet *iso*-PhCl complexes. Values are obtained from natural population analysis. The Ph-Cl bond lengths and the energies of the complexes are also shown.

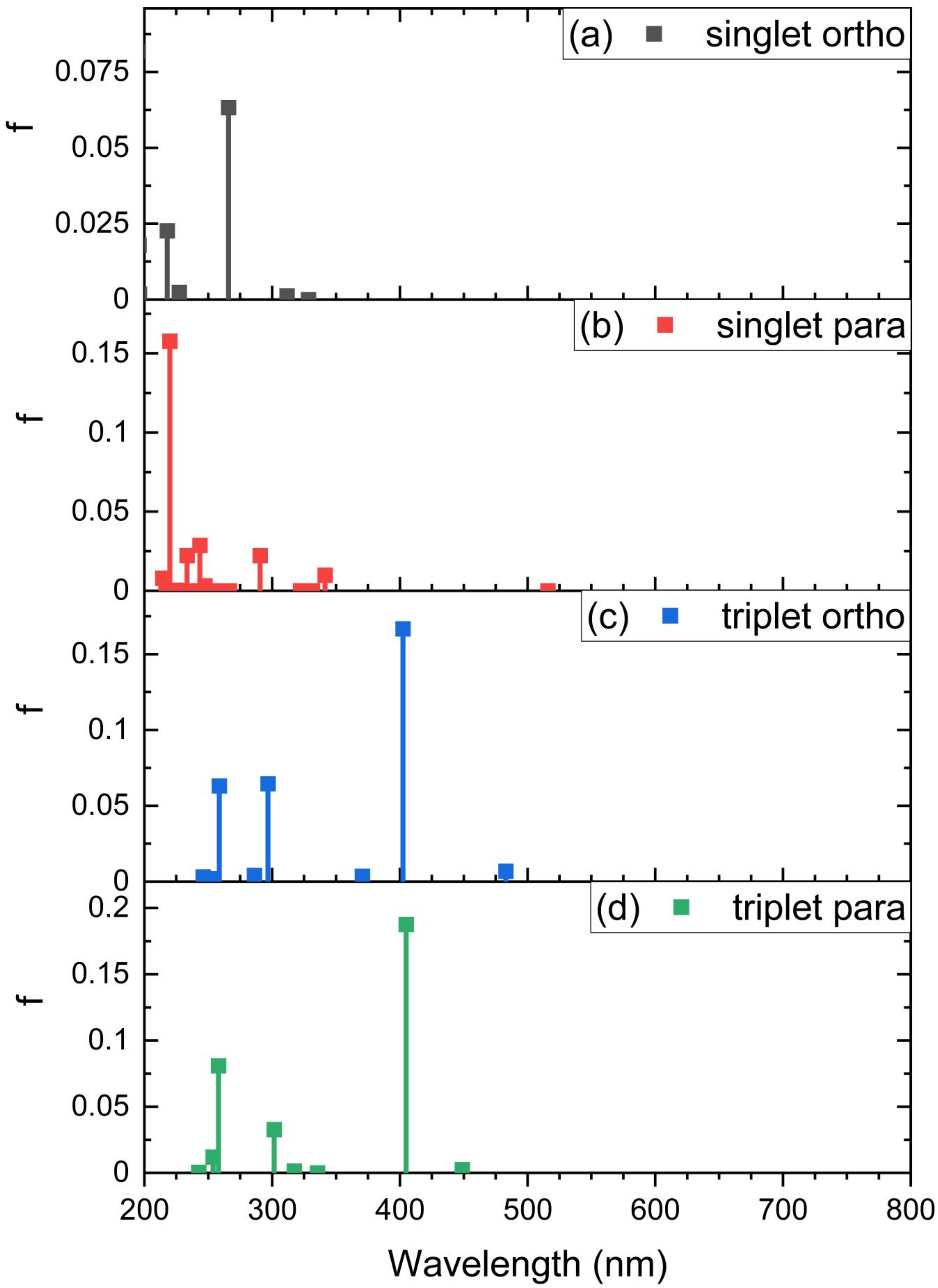

Figure S7. Calculated electronic absorption spectra of *iso*-PhCl complexes in: (a) singlet ortho; (b) singlet para; (c) triplet ortho; and (d) triplet para structures at the TD-DFT CAM-B3LYP/aug-cc-pVTZ level of theory.

TVAS of UV-photoexcited PhCl

The TVA spectra of 270-nm photoexcited PhCl in cyclohexane and perfluorohexane are shown in Figure S8. In these spectra, two negative-going peaks at around 1480 cm^{-1} and 1585 cm^{-1} are assigned to ground state bleaches (GSB) of PhCl because these bands are observed in steady-state FTIR spectra (Figure S8c). The GSB features do not significantly recover in the 5-ns temporal window shown, which suggests only a minor role for geminate recombination of the parent PhCl. Inefficient recombination to the S_0 state of PhCl may be a consequence of homolytic bond cleavage on the T_5 ($n\sigma^*$) PES producing spin-correlated radical pairs with overall triplet spin multiplicity.

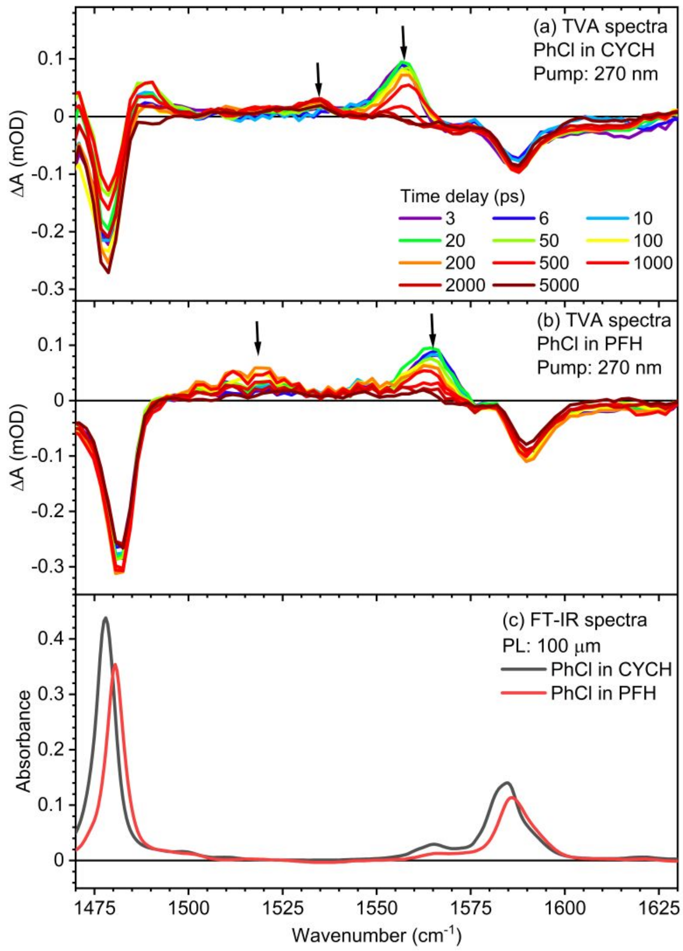

Figure S8. TVA spectra of 270-nm UV-photoexcited PhCl in: (a) cyclohexane; and (b) perfluorohexane. Coloured lines denote spectra obtained at different time delays, as shown in the inset key. Arrows identify the peaks discussed in the main text. (c) Steady-state FTIR spectra of PhCl in cyclohexane and perfluorohexane.

In addition to the GSB features, the transient absorption spectra show a distinct positive peak at around 1560 cm^{-1} and a weaker positive peak at 1520 cm^{-1} in perfluorohexane, or 1535 cm^{-1} in cyclohexane (Figures S8a and S8b). The kinetics of these bands were extracted by fitting with Gaussian basis functions in KOALA.[1] The stronger peaks at ~1560 cm^{-1} in both solvents are assigned to ESA from the S_1 state because their decay kinetics match those obtained from TEAS measurements (Figure S9). In contrast, the kinetics of the peaks at 1535 cm^{-1} in cyclohexane and 1520 cm^{-1} in perfluorohexane suggest intermediate character that rises and decays, with greater intensity in perfluorohexane. Hence, these bands are assigned to the *iso*-PhCl species discussed above. Figure S9 shows the kinetics of the observed bands, fitted with the same sequential reaction model as used for transient electronic absorption spectroscopy data. Offsets are introduced to the fit functions because of the poorer signal-to-noise in the TVAS data, which do not benefit from the large oscillator strengths of the electronic transitions in the triplet-spin *iso*-PhCl species. According to the kinetic analysis shown in Figure 2 in the main text, the *iso*-PhCl decays with a smaller time constant than that for its formation, which severely limits the concentrations produced in our experiments, making the *iso*-PhCl hard to detect. The time constants obtained from fitting the TEA spectra (see Section a in the main text) were therefore used as constraints in these fits.

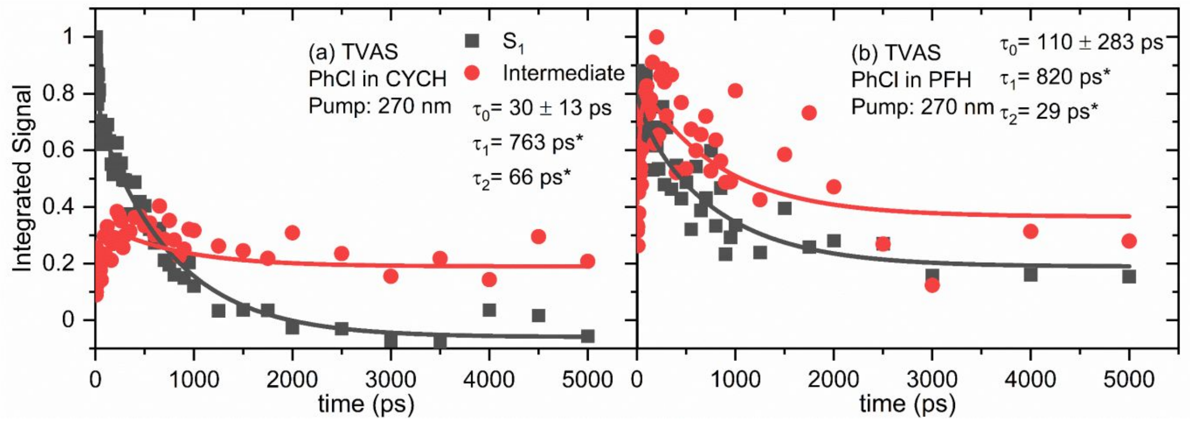

Figure S9. The kinetics of TVAS bands observed at ~1560 cm^{-1}, assigned to a PhCl (S_1) ESA (black squares), and at 1520 / 1535 cm^{-1}, assigned to the photochemical intermediate (red circles) with an IR absorption band at ~1535 cm^{-1}, in (a) cyclohexane, and (b) perfluorohexane. Experimental data (solid symbols) are fitted with a sequential kinetic model (solid lines). In both cases, the fits used time constants fixed to the values obtained from analysis of the TEA spectra (see main text).

A rapid initial decay (represented by the first time constant, τ_0) is attributed to IVR and cooling of the vibrationally hot S_1 molecules prepared by UV absorption. Attempts to confirm proposed assignment of the transient features seen by TVAS draw on the calculated IR absorption spectra of various candidates shown in Figure S10. These calculations, and previous literature, predict no strong IR absorption from phenyl radicals in our probe region.[2] Phenylperoxy radicals are considered because of the possibility of phenyl radical reactions with dissolved oxygen.[3] Based on our calculated IR spectra and previous publications, the phenylperoxyl radical has an expected IR absorption band at ~1480 cm^{-1},[4] where we observe an overlapping PhCl GSB. Comparison of the changes in intensity of the two GSB features at 1480 cm^{-1} and 1585 cm^{-1} should therefore clarify whether phenylperoxy radicals are forming. In cyclohexane, analysis of the 1480 cm^{-1} feature is complicated by an interfering solvent band, but in PFH the recoveries of the two GSB features are similar, which suggests no measurable contribution from absorption by the phenylperoxyl radical within a 5-ns timescale. As Figure S10 shows, triplet ortho and para *iso*-PhCl complexes are predicted to have strong absorption bands at ~1573 cm^{-1} and ~1526 cm^{-1} respectively, with the singlet para complex showing a pair of bands at ~1510 cm^{-1} and ~1540 cm^{-1}. In the TVA spectra in cyclohexane, a transient feature assigned to the intermediate species lies at ~1535 cm^{-1} which may be either the singlet or the triplet para complex. The intermediate peak observed at ~1520 cm^{-1} in perfluorohexane is best assigned to the triplet para complex. This band is broader than other features in the TVA spectra, and so may have a contribution from the singlet para complex as well.

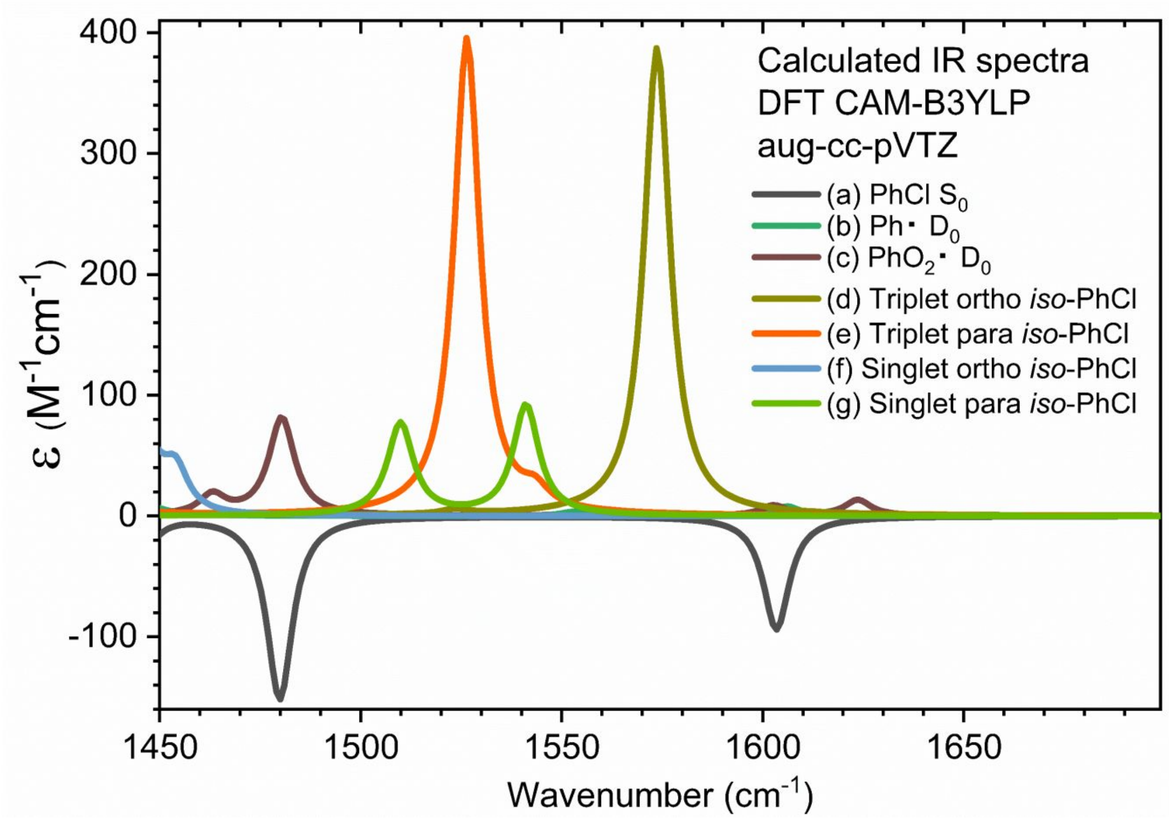

Figure S10. The calculated IR spectra of: (a) chlorobenzene (S_0); (b) phenyl radical; (c) phenylperoxyl radical; (d) triplet ortho *iso*-PhCl; (e) triplet para *iso*-PhCl; (f) singlet ortho *iso*-PhCl; and (g) singlet para *iso*-PhCl, all computed at the DFT CAM-B3LYP/aug-cc-pVTZ level of theory. A wavenumber scaling factor of 0.965 has been applied, and the half-width at half height is set to be 4 cm^{-1}, which is representative of the experimental resolution.

References

1. Grubb, M. P.; Orr-Ewing, A. J.; Ashfold, M. N. R., KOALA: A program for the processing and decomposition of transient spectra. *Rev. Sci. Instrum.* **2014,** *85* (6), 064104.
2. Friderichsen, A. V.; Radziszewski, J. G.; Nimlos, M. R.; Winter, P. R.; Dayton, D. C.; David, D. E.; Ellison, G. B., The Infrared Spectrum of the Matrix-Isolated Phenyl Radical. *J. Am. Chem. Soc.* **2001,** *123* (9), 1977-1988.
3. Da Silva, J. P.; Jockusch, S.; Turro, N. J., Probing the photoreactivity of aryl chlorides with oxygen. *Photochem. Photobiol. Sci.* **2009,** *8* (2), 210-216.
4. Mardyukov, A.; Sander, W., Matrix Isolation and Spectroscopic Characterization of the Phenylperoxy Radical and Its Rearranged Products. *Eur. J. Chem.* **2009,** *15* (6), 1462-1467.
